# Neuropathology of COVID-19: a spectrum of vascular and acute disseminated encephalomyelitis (ADEM)-like pathology

**DOI:** 10.1007/s00401-020-02166-2

**Published:** 2020-05-24

**Authors:** R. Ross Reichard, Kianoush B. Kashani, Nicholas A. Boire, Eleni Constantopoulos, Yong Guo, Claudia F. Lucchinetti

**Affiliations:** 1 Department of Laboratory Medicine and Pathology, Mayo Clinic, Rochester, MN USA; 2 Department of Medicine, Mayo Clinic, Rochester, MN USA; 3 Department of Neurology, Mayo Clinic, Rochester, MN USA

**Keywords:** SARS-CoV-2, COVID-19, Neuropathology, White matter, Infarct, Demyelinating

## Abstract

We report the neuropathological findings of a patient who died from complications of COVID-19. The decedent was initially hospitalized for surgical management of underlying coronary artery disease. He developed post-operative complications and was evaluated with chest imaging studies. The chest computed tomography (CT) imaging results were indicative of COVID-19 and he was subsequently tested for SARS-CoV-2, which was positive. His condition worsened and he died after more than 2 weeks of hospitalization and aggressive treatment. The autopsy revealed a range of neuropathological lesions, with features resembling both vascular and demyelinating etiologies. Hemorrhagic white matter lesions were present throughout the cerebral hemispheres with surrounding axonal injury and macrophages. The subcortical white matter had scattered clusters of macrophages, a range of associated axonal injury, and a perivascular acute disseminated encephalomyelitis (ADEM)-like appearance. Additional white matter lesions included focal microscopic areas of necrosis with central loss of white matter and marked axonal injury. Rare neocortical organizing microscopic infarcts were also identified. Imaging and clinical reports have demonstrated central nervous system complications in patients’ with COVID-19, but there is a gap in our understanding of the neuropathology. The lesions described in this case provide insight into the potential parainfectious processes affecting COVID-19 patients, which may direct clinical management and ongoing research into the disease. The clinical course of the patient also illustrates that during prolonged hospitalizations neurological complications of COVID may develop, which are particularly difficult to evaluate and appreciate in the critically ill.

## Introduction

The coronavirus disease of 2019 (COVID-19) has spread around the world, infecting millions of individuals [3, 12]. Typical clinical presentations of SARS-CoV-2-infected patients, the etiologic agent of COVID-19, include fever, cough, dyspnea, anosmia, and myalgia. COVID-19-associated pathology is gradually being uncovered. Recent postmortem evaluation demonstrates diffuse alveolar damage and acute airway inflammation, which resembles severe acute respiratory syndrome coronavirus (SARS-CoV) and the Middle East respiratory syndrome (MERS-CoV) infection [1, 13]. Recent reports highlight radiographic presentations of COVID-19-associated neuropathologies, including acute hemorrhagic necrotizing encephalitis (AHLE) and acute disseminated encephalomyelitis (ADEM) [11].

Both direct infection, as well as parainfectious mechanisms, may contribute to a spectrum of neuropathological manifestations associated with viral infections. It is well recognized that viral infections may cause demyelinating disease in humans, including such notable examples, as subacute sclerosing panencephalitis from the measles virus and progressive multifocal leukoencephalopathy from the JC virus [4]. Coronavirus has proven to be no exception, with previous non-human primate [9] and murine [2, 7] models demonstrating demyelination in the central nervous system (CNS) following infection. Previous studies have investigated the pathophysiology of SARS-CoV and MERS-CoV infection in the central nervous system (CNS). Isolation of SARS-CoV from infected brain tissue suggests that SARS-CoV is capable of directly infecting the CNS [6, 15]. Additional studies have suggested that angiotensin-converting enzyme 2 (ACE2) protein may be a receptor for SARS-CoV [8] and may be a key to better understanding the pathophysiology of SARS-Cov-2 infections and its complications. ACE2 protein is present in brain endothelium and smooth muscle cells, which raises a potential vascular mechanism of injury. Herein we describe a COVID-19 autopsy case, which illustrates a spectrum of neuropathological findings that include neocortical infarcts, focal hemorrhagic white matter lesions, and discrete foci of acute axonal injury, with associated myelin loss.

## Case report

The decedent was a 71-year-old semi-retired medical professional previously diagnosed with ischemic heart disease due to coronary artery atherosclerosis. He was initially admitted to the hospital for fatigue and exertional dyspnea, consistent with his known heart disease. Coronary angiogram demonstrated mid-left anterior descending artery (LAD) obstruction (70%) along with first obtuse marginal artery (1OM) obstruction (70%). In March 2020, he underwent an elective double coronary artery bypass graft surgery. Six days after the procedure, he developed a left-sided pleural effusion and thoracentesis yielded 450 milliliters of serosanguinous fluid. His respiratory status steadily deteriorated, with worsening symptoms and increasing oxygen demand. Chest radiographs revealed patchy bilateral interstitial abnormalities, with the computed tomography (CT) highlighting parenchymal consolidation and surrounding ground-glass opacities following a peribronchovascular distribution. Nine days after his elective procedure, COVID-19 was suspected, and he subsequently tested positive for SARS CoV-2.

Eleven days after his procedure, the decedent required intubation, sedation/paralysis, and intermittent prone positioning for the management of continued acute respiratory failure. One day prior to his confirmed COVID-19 diagnosis, he developed acute kidney injury (AKI), likely secondary to shock state, respiratory failure, and IV contrast administration. Continuous renal replacement therapy was required for 5 days after AKI diagnosis. He remained critically ill over the following days with a highly inflammatory state, as evidenced by rising levels of CRP, IL-6, and ferritin. Extensive supportive therapies were pursued, including vasopressor support, and stress dose steroids. Despite the initial improvement in gas exchange and lung mechanics, prognosis remained poor, and the family decided on DNR status. The immediate cause of death was attributed to medical treatment-refractory junctional bradyarrhythmia.

## Neuropathological findings

**Fig. 1:**
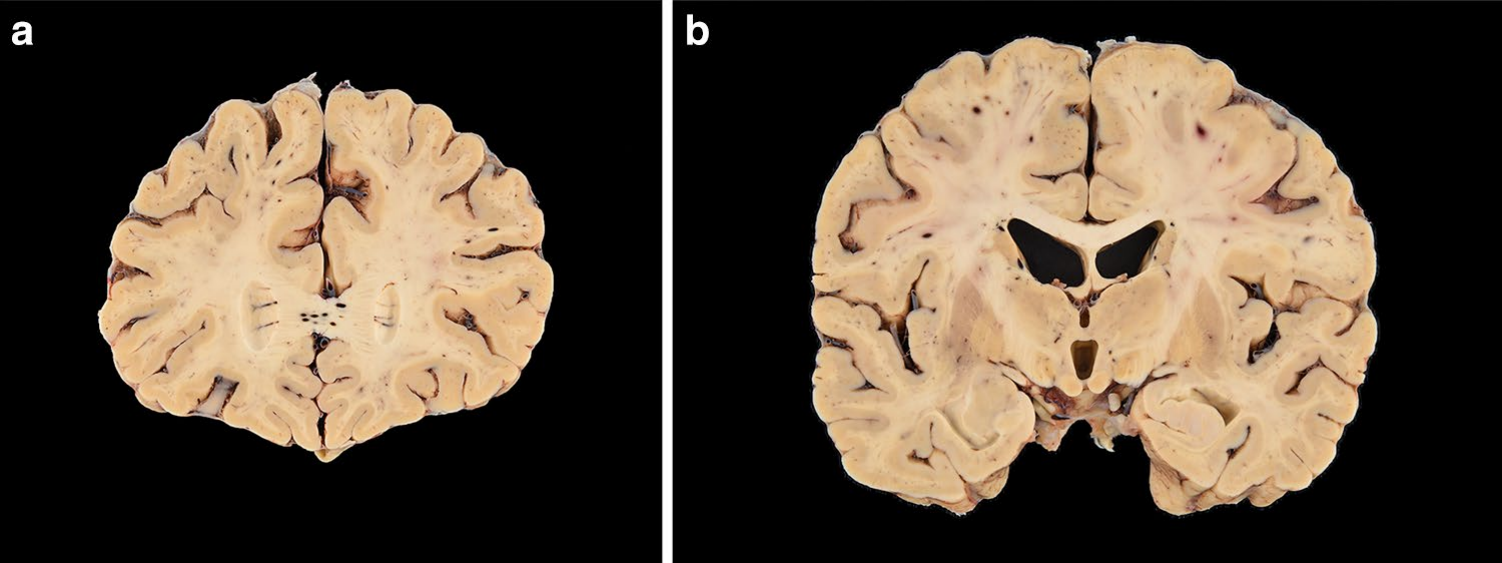
**a** Coronal section of frontal lobes with widespread white matter hemorrhages. **b** Coronal section of cerebral hemispheres with widespread white matter hemorrhages

**Fig. 2:**
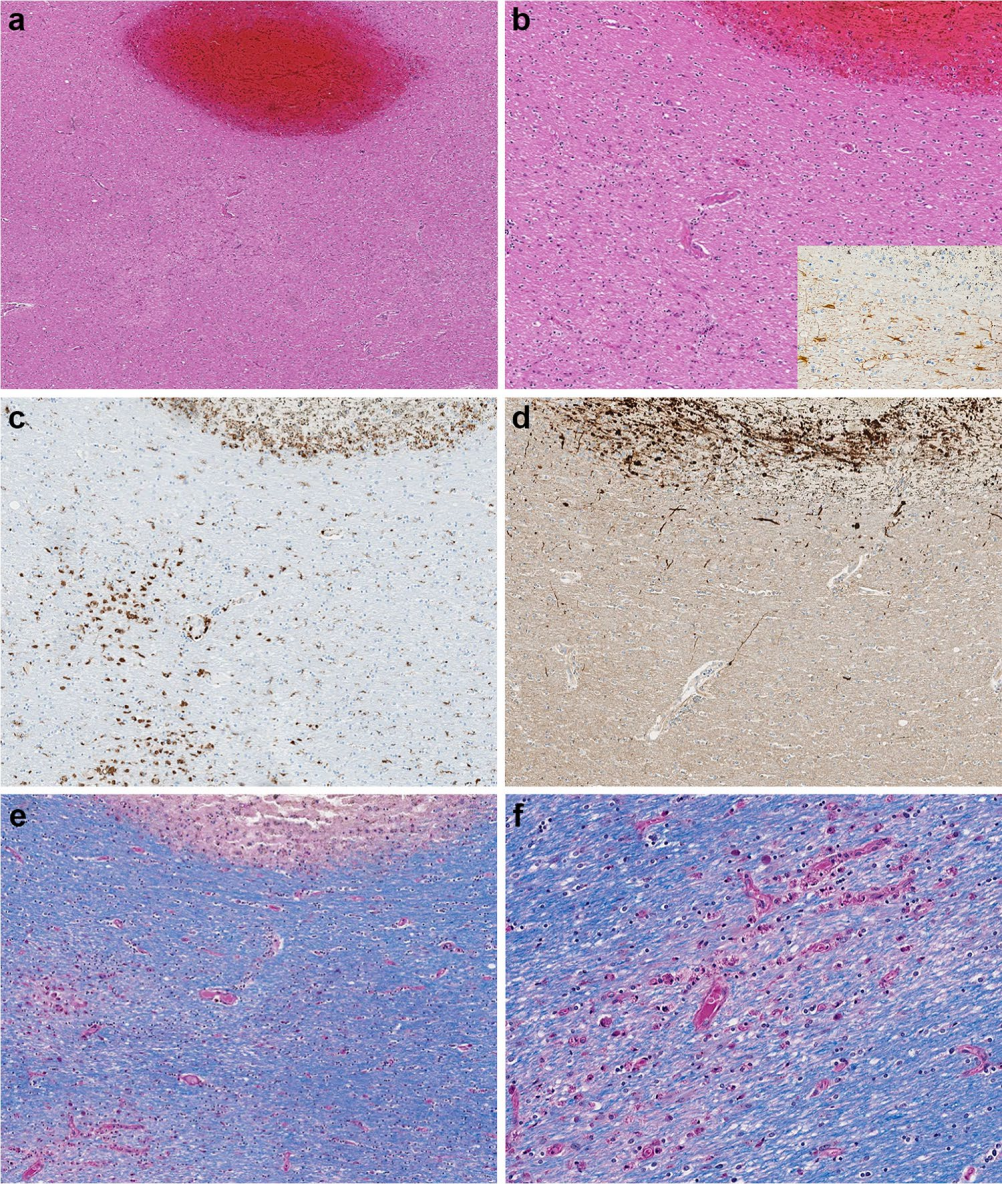
**a**–**d** Microscopic sections of the corpus callosum genu. **a** H&E section of destructive hemorrhagic white matter lesion. **b** H&E section with white matter pallor adjacent to hemorrhagic lesion with GFAP immunoreactive reactive astrocytes evenly distributed in the white matter (inset) **c** CD68 immunostaining highlights a collection of macrophages at the periphery of the hemorrhagic lesion and a macrophages within an area of white matter pallor. **d** APP immunostain identifies axonal swellings within the hemorrhagic lesion and an absence of damaged axons within adjacent region of demyelination. **e**, **f** LFB/PAS stain distinguishes the focal area of myelin loss within the hemorrhagic lesion and adjacent PAS-positive foamy macrophages tracking along blood vessels and (**f**) higher magnification of the perivascular macrophages

The postmortem gross examination of the brain revealed mild brain swelling and hemorrhagic lesions disseminated throughout cerebral hemispheric white matter, ranging in size from 1 mm to 1 cm (Figs. 1a, b and 2a, b). Routine hematoxylin and eosinophilic (H&E) histological examination of these lesions revealed foci of intraparenchymal blood that disrupted the white matter, with macrophages at the periphery of the lesions (Fig. 2a–c), and without significant associated reactive astrogliosis by glial fibrillary acidic protein (GFAP) immunostaining. GFAP immunostains, however, did reveal a widespread background of generalized reactive gliosis within the white matter (Fig. 2b, inset). The amyloid precursor protein (APP) immunostain, a marker of axonal injury, highlighted damaged, swollen axons at the periphery of the hemorrhagic foci (Fig. 2d). Luxol fast blue/periodic acid–Schiff (LFB/PAS), a special stain for myelin, identified loss of myelin, PAS-positive macrophages, and fragmented axonal processes within these lesions (Fig. 2e). A myelin proteolipid protein (PLP) stain also demonstrated remnants of myelin within the lesions, and oligodendrocyte apoptosis surrounding the lesions. Adjacent to the hemorrhagic lesions, the tissue was well preserved without areas of infarct, necrotic blood vessels, or perivascular inflammation.

**Fig. 3:**
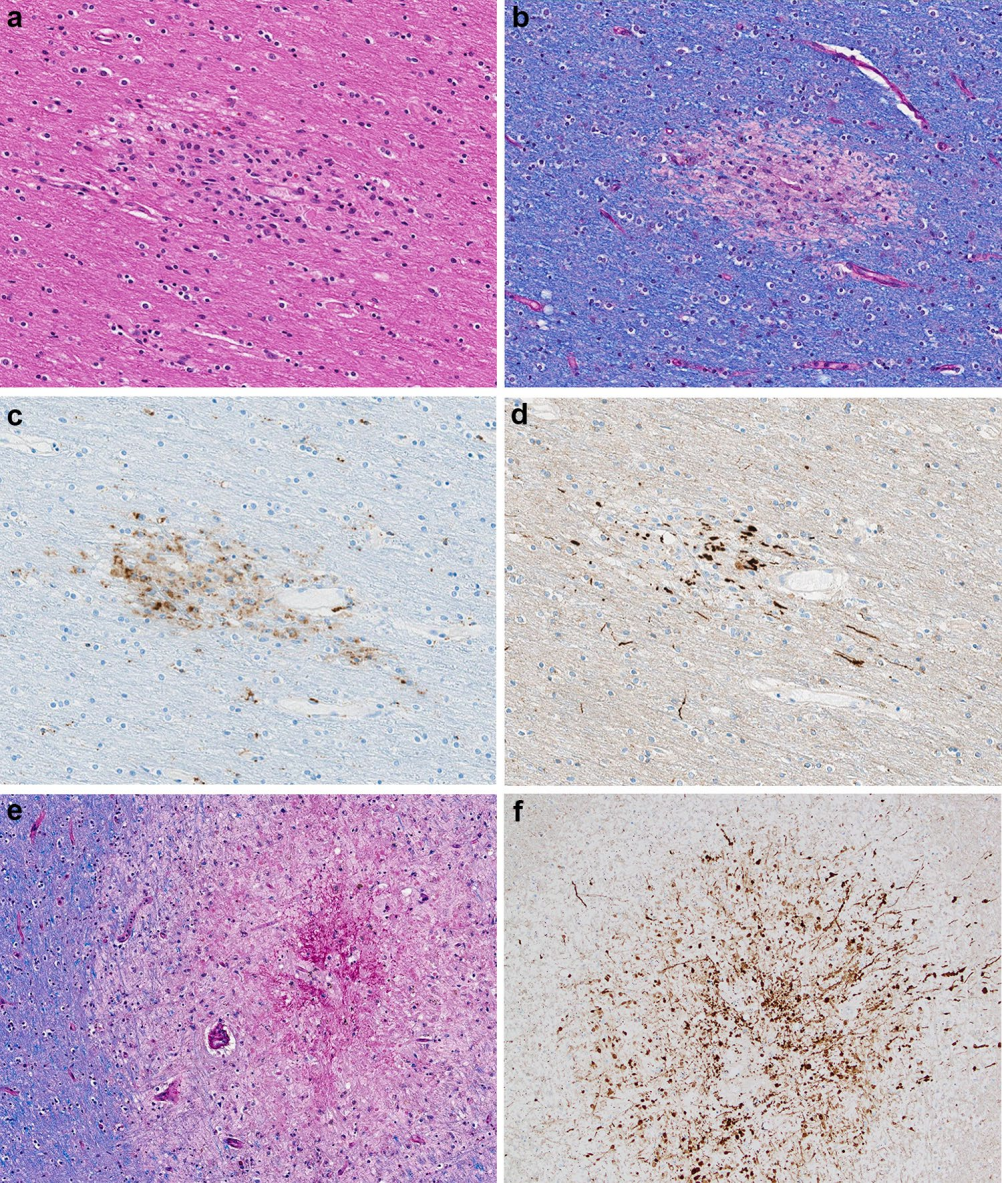
**a** H&E section of subcortical white matter with perivascular pallor. **b** LFB/PAS stained section demonstrates the perivascular myelin loss within the subcortical white matter lesion. **c** CD68 immunostain confirms the perivascular distribution of the macrophages. **d** APP immunostain highlighting some damaged axons present in the region of myelin loss. **e** LFB/PAS-stained section of middle cerebellar peduncle with a central destructive lesion and radiating graduated myelin loss. **f** APP immunostained section of middle cerebellar peduncle lesion highlighting the marked axonal injury

The histologic examination revealed a second type of pathological lesion that on H&E was characterized by subtle areas of subcortical white matter pallor with a variable perivenular distribution (Figs. 2a, b and 3a). A CD68 immunostain of these lesions confirmed the perivascular cellular infiltrates as macrophages (Figs. 2c and 3c) and corresponding myelin loss visible with LFB/PAS stains (Figs. 2e, f and 3b). APP immunostains of these lesions demonstrated a range of axonal injury, from essentially no axonal injury (Fig. 2d) to moderate axonal injury (Fig. 3d). Additional white matter lesions included microscopically identified discrete destructive lesions with central fibrin, associated extravasated red blood cells, and radiating graduated loss of myelin, which were often around a vessel (Fig. 3e). APP immunostains of these lesions highlighted marked axonal injury (Fig. 3f).

**Fig. 4:**
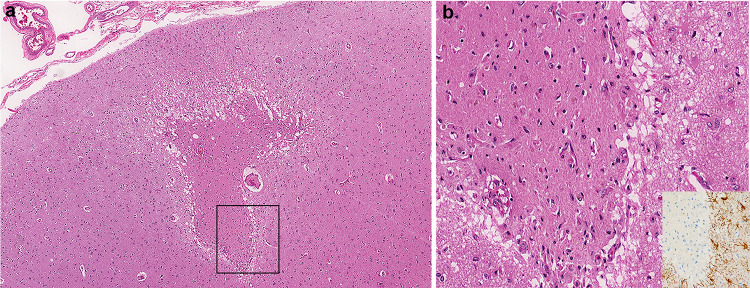
**a** Microscopic H&E section of frontal lobe cortex with organizing infarct. **b** Higher magnification of the infarct with reactive blood vessels and adjacent GFAP immunoreactive reactive astrocytes (inset)

A few microscopic cortical organizing/organized infarcts were also identified that were not appreciable on gross examination (Fig. 4). GFAP immunostains confirmed the presence of a rim of astrogliosis surrounding the infarcts (Fig. 4b insert). PLP staining demonstrated preservation of subpial myelin in the adjacent intact cortex. The neocortex did have scattered necrotic neurons, as did the hippocampus (CA1 region), and cerebellum (Purkinje cells), indicative of terminal hypoxic–ischemic injury. Regional infarcts, however, were not present in the brain, brainstem, or spinal cord. Of note, lymphocytic inflammation was not present in the leptomeninges and was minimally present surrounding blood vessels, confirmed with immunostains. A CD3 immunostain of the neocortex in the region of the cortical infarct highlighted a few T cells within, and adjacent to, the infarct and confirmed the absence of perivascular and leptomeningeal infiltrates. A CD20 immunostain of the aforementioned region revealed no B cell activation. CD3 immunostains of subcortical white matter with both destructive hemorrhagic lesions and lesions with relative preservation of the white matter, highlighted scant perivascular T cells, evenly distributed throughout the section, and an absence of CD20-positive B cells. Scattered granulocytes were present in the destructive white matter lesions, however. In contrast to the white matter, the deep grey nuclei, brainstem, and spinal cord, did not have any apparent pathological processes. Interestingly, microscopic lesions were present in the internal capsule with unremarkable adjacent deep grey nuclei, including the absence of neuronal necrosis. No microglial nodules (H&E and GFAP immunostains) or evidence of neuronophagia was present in the brainstem, spinal cord, or deep grey nuclei. CD68 immunostains of the brainstem highlighted perivascular pigment-laden macrophages associated with arteriolosclerosis in the context of otherwise normal structures (H&E and LFB/PAS stains). Routine histological examination of the olfactory bulb/nerve revealed only aging-related corpora amylacea.

## Discussion

Our understanding of SARS CoV-2 infections and COVID-19 is rapidly evolving; significant gaps in our knowledge remain. The neuropathology in the case presented raises questions about the pathophysiology of the infection in the central nervous system (CNS) and provides insights into the neurological complications of these patients. Parainfectious conditions are well known to develop after viral infections, including SARS. In this case, the pathological lesions are distinct and the interpretation is not confounded by underlying severe global hypoxic–ischemic brain injury with concomitant regional infarcts. The striking features of the pathology presented include the range of subcortical white matter pathology, the absence of deep grey nuclei or brainstem pathology, and the lack of typical features of viral and post-viral encephalitides.

We describe several types of pathological lesions that may contribute to neurological manifestations of COVID-19 patients. The widespread hemorrhagic white matter lesions identified have some of the characteristic features of AHLE. Similar hemorrhagic lesions have previously been described with SARS-CoV, but are reported within a background of extensive neuronal necrosis [15], which we did not observe. AHLE often presents fulminantly and results in rapid death. The relative paucity of significant reactive changes in the case presented suggests that, indeed, these occurred in close chronological proximity to the patient’s death. Clearly necrotic blood vessels and perivascular inflammation, however, were not identified in association with the larger hemorrhagic lesions. The prominent acute axonal injury and loss, coupled with preserved remnants of myelin within these hemorrhagic lesions, is also not typical of classic AHLE. Another distinct pathology identified was characterized by small (< 5 mm) white matter lesions with clusters of macrophages and a range of associated axonal injury. Some lesions appeared to be more perivenular in nature, tracking along vessels, resembling an acute disseminated encephalomyelitis (ADEM)-like histological appearance. However, the variable perivenular association, coupled with the absence of intracortical and cortical subpial demyelination, absence of cortical microglial activation, paucity of lymphocytic inflammation, and prominent acute axonal injury are not characteristic of classic ADEM [16]. Although not all lesions were clearly necrotic, it is also possible these white matter lesions are vascular in origin. Other lesions had marked central axonal injury, associated extravasated blood, and surrounding myelin loss, reminiscent of subtle “ball-and-ring” microhemorrhages. Whether these white matter lesions reflect distinct pathological processes or a continuum of a single disease is unclear. The neocortical microscopic infarcts identified raise the possibility of microthromboembolic events, possibly related to COVID-19 and associated complications, or in this patient, could be related to his other underlying medical conditions and treatments.

The fundamental question is whether the observed neuropathological lesions are a result of primary vascular disease with secondary white matter injury (e.g. acute axonal injury, secondary demyelinating pathology) or is due to a parainfectious primary demyelinating type of disease or a combination. Parainfectious conditions, such as post-viral autoimmune disorders, have been described in association with a variety of viruses, including coronaviruses (e.g., SARS-CoV), and variety of potential routes of CNS infection have been proposed [15]. The potential explanations of the neurological symptoms include direct infection (e.g. viral encephalitis), metabolic etiologies of encephalopathy, and secondary cerebrovascular insults [14]. Imaging case studies of COVID-19 patients have suggested pathological lesions characteristic of ADEM, and acute hemorrhagic AHLE [11]. Recent descriptions of large vessel cerebral infarcts in patients that would not be expected based upon their age and co-morbidities suggest an underlying vasculopathy or coagulopathy [10]. Research into the pathophysiology of SARS-CoV proposes a role for the viral ACE2 receptor as a potential mechanism of injury [5]. The lack of microglial nodules, the paucity of perivascular and leptomeningeal inflammation, absence of neuronophagia, and preservation of deep gray structures/brainstem/spinal cord distinguish it from other viral encephalitides and would suggest a vascular origin with secondary myelin loss.

Parainfectious neuropathological processes typically present after a latent period following an infectious illness. The prolonged course of this patient suggests that during his hospitalization, he developed a secondary disease initiated by SARS CoV-2, which raises the clinical question of when, or if, to consider evolving neurological processes during a prolonged period of intubation. Further complicating the clinical management of these patients is the microscopic white matter lesions, and cortical infarcts would not be readily apparent with routine imaging studies. Furthermore, these patients may not be stable, or appropriate, for transporting for more complex imaging studies. Our understanding of the parainfectious neuropathology of SARS CoV-2 continues to evolve, but remains a cause of either devastating outcome or morbid sequelae in many patients that do survive.
